# Long-term outcome of tailored antithrombotic therapy based on platelet function testing in patients undergoing percutaneous coronary intervention: a 5-year retrospective cohort study

**DOI:** 10.1007/s00380-025-02584-w

**Published:** 2025-08-21

**Authors:** Emanuele Cecchi, Andrea Grasso Granchietti, Claudia Assenza, Angela Ilaria Fanizzi, Manuel Garofalo, Francesca Maria Di Muro, Veronica Speranza Vitiello, Francesco Losanno, Sabina Caciolli, Chiara Piazzai, Marco Chiostri, Rossella Marcucci

**Affiliations:** 1 Department of Clinical and Experimental Medicine, University of Florence, School of Human and Health Science, Largo Brambilla 3, Florence, Italy; 2 General Cardiology Unit, Department of Cardiac Thoracic and Vascular Medicine, Azienda Ospedaliero-Universitaria Careggi, Florence, Italy; 3 Department of Medicine, Surgery and Dentistry, University of Salerno, Baronissi, Salerno Italy; 4 Center for Atherothrombotic Disease, Department of Experimental and Clinical Medicine, University of Florence, Florence, Italy

**Keywords:** Tailored antithrombotic therapy, Platelet function test, Percutaneous coronary intervention, Dual antiplatelet therapy

## Abstract

Dual antiplatelet therapy is the standard therapy for the secondary prevention of acute and chronic coronary syndromes in patients undergoing percutaneous coronary intervention (PCI). The introduction of more potent antiplatelet agents and understanding of prognostic implications associated with bleeding have led to a substantial evolution in antiplatelet treatment regimens over the past decades. Several investigations have been conducted to better stratify patients undergoing PCI according to their ischemic and bleeding risks and to optimize antithrombotic regimens accordingly. One of the available strategies involves using platelet aggregation tests to determine the most suitable antiplatelet agent to combine with aspirin. Our aim was to evaluate the role of platelet function tests (PFT) in clinical practice in choosing dual antiplatelet therapy for patients undergoing PCI: in this study, we compared the impact on ischemic and hemorrhagic cardiovascular events in a 5 year follow-up between patients treated according to standard guidelines and those treated with a platelet function test guided approach. This study included 490 patients with acute or chronic coronary syndrome who underwent percutaneous angioplasty between 2013 and 2016 and were subsequently treated with dual antiplatelet therapy. Patients whose treatment strategy was based on PFT were 68.4% (*n* = 335), while others received standard therapy. The primary endpoint of the study was to assess the incidence of net adverse clinical events (NACE), defined as a composite of all-cause mortality, myocardial infarction, stroke, or major bleeding, according to the BARC scale. Follow-up was conducted 5 years after angioplasty by telephone contact or by consulting patients’ medical records. Heart failure and stable angina were considered as secondary endpoints. From the univariate analysis, the incidence of NACE was significantly lower in patients who received tailored therapy (33.7% vs. 43.9% in the non-tailored group, *p* = 0.02). In addition, results showed that total length of implanted stents and left main coronary disease were independent risk factors for net adverse clinical events (NACE). Similarly, an initial diagnosis of N-STEMI or unstable angina was associated with an increased risk of adverse events during follow-up. In patients undergoing PCI, a tailored antithrombotic approach guided by PFT appears safe and effective, may represent a feasible strategy in contemporary practice and should be considered in case of high bleeding risk.

**Graphical Abstract:**

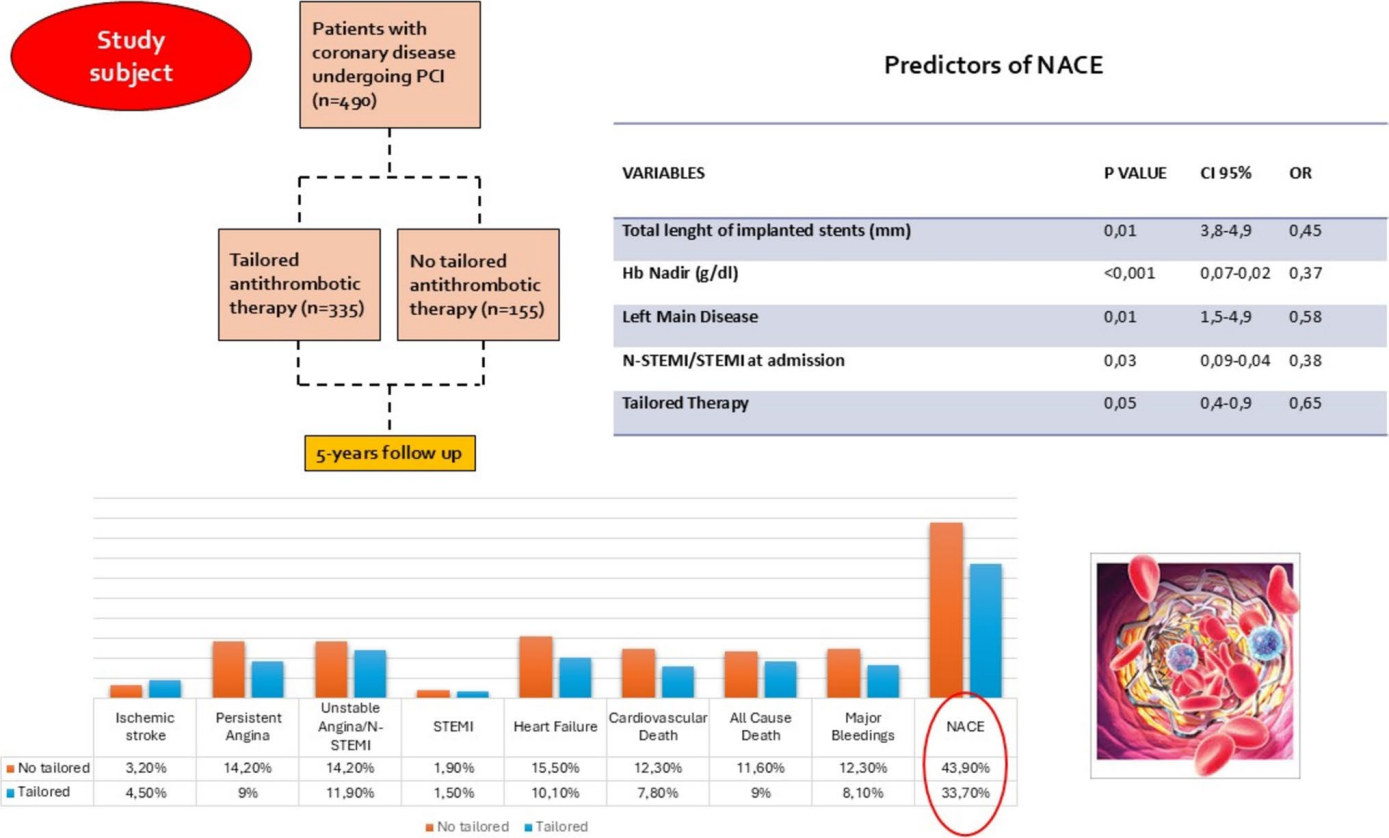

## Introduction

Percutaneous coronary intervention (PCI) has become the cornerstone of revascularization strategies in patients with coronary artery disease (CAD). However, the dual risk of ischemic and hemorrhagic complications poses significant clinical challenges, particularly in the context of long-term outcomes. Optimizing antithrombotic therapy to minimize both ischemic and bleeding events is crucial, especially in high-risk populations such as those presenting with acute coronary syndromes (ACS), including ST-segment elevation myocardial infarction (STEMI) and non-STEMI (N-STEMI) [1, 2]. In the past twenty years, new P2Y12 inhibitors, such as prasugrel and ticagrelor, have been developed, offering greater potency and less influence from CYP450 cytochrome genetic polymorphisms compared to clopidogrel [3–5]. Their gradual introduction has led to a decrease in the incidence of thrombotic events in patients who have undergone PCI. However, due to their increased antiplatelet potency, these innovative drugs also intensify the risk of hemorrhagic events [6]. This is mostly due to the variability of platelet reactivity in response to therapy, evaluated either through platelet function testing (PFT) [7] or pharmacogenomic approaches [8] and emerged as key determinant of clinical outcomes following PCI [9]. These profiles, along with a deeper understanding of individual variability in response to antiplatelet agents, have led to the development of personalized treatment regimens tailored to patient-specific characteristics [10]. Although the role of tailored therapy remains controversial, several studies have demonstrated its efficacy in reducing both ischemic events and bleeding complications in patients undergoing PCI [11]. Nevertheless, long-term data on its effectiveness remain scarce. The aim of our study was to assess the long-term outcomes of tailored antithrombotic therapy based on platelet function in a real-world cohort of patients undergoing PCI.

## Material and methods

### Study design and population

This is a single-center, retrospective study conducted on 490 consecutive patients who underwent PCI between 2013 and 2016. We included patients with documented coronary artery disease who received stent implantation. All participants were stratified according to the use of tailored antithrombotic therapy, guided by PFT (Light Transmittance Aggregometry with ADP10-PA), compared to those receiving standard therapy. Exclusion criteria were as follows: patients without follow-up data, patients with known hypersensitivity or contraindications to antiplatelet therapy, patients with active bleeding disorders (e.g., hemophilia, thrombocytopenia), or a history of major bleeding events unrelated to PCI or antithrombotic therapy. Follow-up was conducted by telephone contact or by consulting patients’ medical records; data were collected for five years to assess net adverse clinical events (NACE), defined as the composite of cardiovascular death, non-fatal myocardial infarction, non-fatal stroke, and non-fatal major bleeding (according to BARC bleeding score) [12].

Arterial hypertension was defined as a documented history of hypertension or the use of antihypertensive medications at the time of hospitalization. Hypercholesterolemia was defined as total cholesterol levels ≥ 200 mg/dL or low-density lipoprotein cholesterol ≥ 130 mg/dL, or current treatment with lipid-lowering therapy. Obesity was defined as a body mass index (BMI) ≥ 30 kg/m^2^. Atrial fibrillation (AF) included both paroxysmal and persistent AF, as documented in the patient’s clinical history or observed during hospitalization. AF was defined by the presence of irregular rhythm with absence of P waves lasting more than 10 s at 12-lead electrocardiogram (ECG) or 30 s at 3-leads continuous rhythm monitoring. Peripheral artery disease (PAD) was defined as a history of intermittent claudication, previous limb revascularization, or an ankle-brachial index (ABI) < 0.9. Chronic kidney disease (CKD) was defined as an estimated glomerular filtration rate (eGFR) < 60 mL/min/1.73 m^2^ at baseline. Left Ventricular Ejection Fraction was calculated according to the Simpson’s rule, from the apical two and four-chamber views. Unstable angina (UA) was characterized by new-onset or worsening chest pain at rest or with minimal exertion, without elevation of cardiac biomarkers. N-STEMI was diagnosed based on the presence of elevated cardiac biomarkers (troponin) without ST-segment elevation on ECG. STEMI was defined as persistent ST-segment elevation ≥ 1 mm in at least two contiguous leads on ECG, along with elevated cardiac biomarkers. Stable angina was diagnosed in patients with exertional chest pain relieved by rest or nitroglycerin, without evidence of acute plaque rupture or myocardial necrosis. Left main disease was defined as a ≥ 50% stenosis in the left main coronary artery on coronary angiography. Complete revascularization was achieved when all significant coronary stenoses (≥ 50%) in major epicardial vessels were treated. High platelet reactivity (HPR) was defined as an ADP10-PA ≥ 70% on LTA.

This study was conducted in accordance with the Declaration of Helsinki and approved by the Ethics Committee of Careggi University Hospital (CEAVC 25879).

### Tailored therapy protocol

Patients in the tailored therapy group underwent platelet function testing within 48 h after PCI. Based on platelet reactivity, therapy adjustments were made, with a particular focus on switching between P2Y12 inhibitors (clopidogrel, prasugrel, or ticagrelor). Standard therapy patients received P2Y12 inhibitors according to established guidelines of that era [13, 14] without adjustment based on platelet reactivity. All patients received aspirin (100 mg once daily) as part of dual antiplatelet therapy. In our institution, the decision to perform PFT was at the clinical cardiologist’s discretion. This is reflected in the lack of significant differences between the two groups (tailored and no tailored) in the main clinical, biohumoral, and angiographic/procedural features except few variables (see Results section and Table 1). In patients undergoing platelet function testing, platelet reactivity to aspirin was also assessed using arachidonic acid-induced aggregation, but this result did not influence the clinical decision and no treatment changes were made based on aspirin reactivity.

**Table 1:** Clinical, procedural, biochemical, and follow-up data comparison between the no tailored and tailored group

Variable	No tailored (*n* = 155)	Tailored (*n* = 335)	*P* value
Age, mean (SD), y	72.5 (± 10.5)	68.6 (± 11.7)	0.07
Sex, No. (%)			
Male, No. (%)	121 (78.1%)	259 (77.3%)	0.5
Female, No. (%)	34 (21.9%)	76 (22.7%)	0.5
BMI, mean (SD), Kg/m2	28.9 (± 5.2)	26.2 (± 3.7)	0.09
Cardiac risk factors			
Family history of CAD, No. (%)	44 (28.6%)	95 (28.4%)	0.5
Former smoker, No. (%)	105 (66.7%)	241 (71.9%)	0.3
Diabetes Mellitus, No. (%)	41 (26.5%)	82 (24.5%)	0.3
Arterial Hypertension, No. (%)	120 (77.4%)	236 (70.4%)	0.06
Hypercholesterolemia, No. (%)	107 (69%)	213 (63.6%)	0.1
Obesity, No. (%)	25 (16.1%)	47 (14%)	0.3
AF, No. (%)	24 (15.5%)	22 (6.6%)	0.003
PAD, No. (%)	30 (19.4%)	41 (12.2%)	0.02
CKD, No. (%)	18 (11.3%)	30 (8.7%)	0.2
Previous AMI, No. (%)	38 (24%)	63 (18.9%)	0.2
Previous PCI, No. (%)	44 (28.4%)	74 (22.2%)	0.08
Previous CABG, No. (%)	15 (9.7%)	11 (3.3%)	0.004
LVEF, mean (SD), %	50.8% (± 10.2)	51.4% (± 9.5)	0.08
Patient’s presentation			
Unstable angina, No. (%)	42 (27.3%)	87 (26%)	0.5
N-STEMI, No. (%)	38 (24.7%)	81 (24.2%)	0.5
STEMI, No. (%)	32 (20.8%)	113 (33.7%)	0.005
Stable angina, No. (%)	42 (27.2%)	54 (16.1%)	0.005
Arterial access			
Radial, No. (%)	120 (77.4%)	266 (79.4%)	0.3
Femoral, No. (%)	35 (22.6%)	69 (20.6%)	0.3
Antiplatelet therapy before/in cath lab, No. (%)	70 (61.9%)	157 (62.5%)	0.9
Left main disease, No. (%)	18 (11.6%)	33 (9.9%)	0.3
Treated vessels			
1, No. (%)	98 (63.2%)	216 (64.5%)	0.8
2, No. (%)	43 (27.7%)	89 (26.6%)	0.8
≥ 3, No. (%)	14 (9.1%)	30 (8.9%)	0.8
Implanted stents			
0, No. (%)	8 (1.6%)	8 (1.6%)	0.1
1, No. (%)	78 (49.4%)	152 (45%)	0.1
2, No. (%)	39 (25.3%)	95 (28.7%)	0.1
≥ 3, No. (%)	31 (20%)	79 (23.6%)	0.1
Total length of implanted stents, mean (SD), mm	33.5 (± 23.4)	33 (± 18.8)	0.02
Complete revascularization, No. (%)	88 (56.8%)	172 (51.3%)	0.5
Blood tests			
Hb at admission, mean (SD), g/dl	13.1 (± 1.9)	13.6 (± 1.8)	0.2
Post-operative Hb (lowest), mean (SD), g/dl	11.7 (± 2.1)	11.8 (± 1.9)	0.1
Platelets ad admission, mean (SD), n/μl	209,530 (± 77,259)	217,100 (± 65,252)	0.6
Post-operative platelets (lowest), mean (SD), n/μl	171,780 (± 60,839)	180,260 (± 52,273)	0.6
eGFR, mean (SD), dl/min	73.4 (± 44.5)	75.3 (± 41.1)	0.5
INR, mean (SD)	1.4 (± 0.6)	1.3 (± 0.4)	< 0.001
Upgrade to ticagrelor, No. (%)	14 (9%)	30 (9%)	0.5
Upgrade to prasugrel, No. (%)	8 (5.2%)	10 (3%)	0.2
Downgrade to clopidogrel, No. (%)	0 (0%)	5 (1.5%)	0.1
DAT at discharge, No. (%)	23 (14.8%)	20 (6%)	0.002
5-year events			
Ischemic stroke, No. (%)	5 (3.2%)	15 (4.5%)	0.3
Persistent angina, No. (%)	22 (14.2%)	30 (9%)	0.06
Unstable angina/N-STEMI, No. (%)	22 (14.2%)	40 (11.9%)	0.3
STEMI, No. (%)	3 (1.9%)	5 (1.5%)	0.5
Heart failure, No. (%)	24 (15.5%)	34 (10.1%)	0.06
Cardiovascular death, No. (%)	19 (12.3%)	26 (7.8%)	0.2
All-cause death, No. (%)	18 (11.6%)	30 (9%)	0.2
Major bleedings, No. (%)	19 (12.3%)	27 (8.1%)	0.03
NACE, No. (%)	68 (43.9%)	113 (33.7%)	0.02

*BMI* Body mass index, *CAD* Coronary artery disease, *AF* Atrial fibrillation, *PAD* Peripheral artery disease, *CKD* Chronic kidney disease, *AMI* Acute myocardial infarction, *PCI* Percutaneous coronary intervention, *CABG* Coronary artery bypass grafting, *LVEF* Left ventricular ejection fraction, *N-STEMI* Non-ST-segment elevation myocardial infarction, *STEMI* ST-segment elevation myocardial infarction, *eGFR* estimated glomerular filtration rate, *INR* International normalized ratio, *HPA* High platelet reactivity, *AA* Arachidonic acid, *DAT* Dual antithrombotic therapy (oral anticoagulation and antiplatelet therapy), *DAPT* Dual antiplatelet therapy, *NACE* Net adverse cardiovascular events

### Statistical analysis

Statistical analysis was performed using software SPSS (IBM, USA, version 29.0.2.0). All variables showing Gaussian distribution are displayed as mean values + standard deviation (SD). All continuous variables were analyzed using Student’s *t*–test. Categorical data were summarized as numbers and percentages. Categorical data were compared using Pearson’s ^2^ test and Fisher Exact test when appropriate. Statistical significance was set at a *p* value < 0.05. Univariate and multivariate logistic regression analyses were utilized to assess the relationship between different variables and the occurrence of the primary outcome (NACE at five years). Secondary outcomes included individual components of the NACE composite, angina, and heart failure.

## Results

### Baseline characteristics

Clinical, procedural, biohumoral, and follow-up features of overall patients are described in Table 2. A total of 490 patients were included in this study, with 335 (68.4%) receiving tailored therapy based on platelet function testing. In the tailored therapy group, at discharge, 61.3% of patients were prescribed clopidogrel, 28.7% ticagrelor, and 10% prasugrel. In the standard therapy group, clopidogrel was used in 78.1% of cases, ticagrelor in 16.1%, and prasugrel in 5.8%. A small number of therapy changes occurred in the standard group due to clinical reasons (e.g., clinical or procedural characteristics indicating a high thrombotic risk or side effects), not guided by PFT. Among tailored patients, the use of ticagrelor or prasugrel was more frequent in those with high platelet reactivity (ADP10-PA ≥ 70%). Baseline characteristics were well matched between the two groups, with a median age of 65 years and 26.5% of patients presenting with diabetes. Approximately 32% of patients had left main disease, and 42% presented with STEMI/N-STEMI at admission. In our study population, the overall incidence of NACE at 5-year follow-up was 36.9%, with a detailed breakdown as follows: ischemic stroke occurred in 4.1% of patients, persistent angina in 10.6%, N-STEMI/UA in 12.7%, STEMI in 1.6%, heart failure in 11.8%, cardiovascular death in 9.2%, all-cause death in 9.8%, and major bleedings in 9.4%.

**Table 2:** Clinical, procedural, biochemical, and follow-up data of overall patients

Variable	Overall patients (*n* = 490)
Age, mean (SD), y	69.8 (± 11.5)
Sex, No. (%)	
Male, No. (%)	380 (77.6%)
Female, No. (%)	110 (22.4%)
BMI, mean (SD), Kg/m2	26.1 (± 4.2)
Cardiac risk factors	
Family history of CAD, No. (%)	141 (28.8%)
Former/active smokers, No. (%)	346 (70.6%)
Diabetes Mellitus, No. (%)	123 (25.1%)
Arterial Hypertension, No. (%)	356 (72.7%)
Hypercholesterolemia, No. (%)	320 (65.3%)
Obesity, No. (%)	72 (14.7%)
AF, No. (%)	46 (9.4%)
PAD, No. (%)	71 (14.5%)
CKD, No. (%)	48 (9.8%)
Previous AMI, No. (%)	101 (20.6%)
Previous PCI, No. (%)	118 (24.1%)
Previous CABG, No. (%)	27 (5.5%)
LVEF, mean (SD), %	51.2% (± 9.7)
Patient’s presentation	
Unstable Angina, No. (%)	130 (26.5%)
N-STEMI, No. (%)	119 (24.3%)
STEMI, No. (%)	145 (29.6%)
Stable Angina, No. (%)	96 (19.6%)
Arterial access	
Radial, No. (%)	386 (78.8%)
Femoral, No. (%)	104 (21.2%)
Antiplatelet therapy before/in Cath Lab, No. (%)	227 (62.4%)
Left Main Disease, No. (%)	51 (10.4%)
Treated vessels	
1, No. (%)	314 (64%)
2, No. (%)	132 (26.2%)
≥ 3, No. (%)	44 (9.8%)
Number of implanted stents	1.9 (± 1.3)
0, No. (%)	16 (3.3%)
1, No. (%)	230 (46.9%)
2, No. (%)	134 (27.4%)
≥ 3, No. (%)	110 (22.4%)
Total length of implanted stents, mean (SD), mm	33.2 (± 20.6)
Complete revascularization, No. (%)	260 (53%)
Hemorrhagic events during hospitalization, No. (%)	10 (2%)
Death during hospitalization, No. (%)	7 (1.4%)
Blood tests	
Hb at admission, mean (SD), g/dl	13.4 (± 1.8)
Post-operative Hb (lowest), mean (SD), g/dl	11.8 (± 1.9)
Platelets ad admission, mean (SD), n/μl	214,710 (± 69,285)
Post-operative platelets (lowest), mean (SD), n/μl	177,570 (± 55,205)
eGFR, mean (SD), dl/min	74.7 (± 42.1)
INR, mean (SD)	1.3 (± 0.5)
ADP10-PA test	
HPA (≥ 70%), No. (%)	88 (18%)
Responder (< 70%), No. (%)	319 (65.1%)
Not performed, No. (%)	83 (16.9%)
AA test	
HPA (≥ 20%), No. (%)	112 (22.9%)
Responder (< 20%), No. (%)	295 (60.2%)
Not performed, No. (%)	83 (16.9%)
Tailored Therapy, No. (%)	335 (68.4%)
Ticagrelor as initial therapy, No. (%)	139 (27.7%)
Upgrade to ticagrelor, No. (%)	44 (9%)
Upgrade to prasugrel, No. (%)	18 (3.7%)
Downgrade to clopidogrel, No. (%)	5 (1%)
DAT at discharge, No. (%)	43 (8.8%)
DAPT duration, mean (SD), months	15 (± 3)
5-year events	
Ischemic stroke, No. (%)	20 (4.1%)
Persisting angina, No. (%)	52 (10.6%)
Unstable angina/N-STEMI, No. (%)	62 (12.7%)
STEMI, No. (%)	8 (1.6%)
Heart Failure, No. (%)	58 (11.8%)
Cardiovascular Death, No. (%)	45 (9.2%)
All-Cause Death, No. (%)	48 (9.8%)
Major Bleedings, No. (%)	46 (9.4%)
NACE, No. (%)	181 (36.9%)
Distance of NACE from PCI, mean (SD), months	51.8 (± 17.5)

*BMI* Body mass index, *CAD* Coronary artery disease, *AF* Atrial fibrillation, *PAD* Peripheral artery disease, *CKD* Chronic kidney disease, *AMI* Acute myocardial infarction, *PCI* Percutaneous coronary intervention, *CABG* Coronary artery bypass grafting, *LVEF* Left ventricular ejection fraction, *N-STEMI* Non-ST-segment elevation myocardial infarction, *STEMI* ST-segment elevation myocardial infarction, *eGFR* estimated glomerular filtration rate, *INR* International normalized ratio, *HPA* High platelet reactivity, *DAT* Dual antithrombotic therapy (oral anticoagulation and antiplatelet therapy), *DAPT* Dual antiplatelet therapy, *NACE* Net adverse cardiovascular events

Patients were divided in two groups, tailored and no tailored. Notably, the non-tailored group exhibited a higher prevalence of comorbid conditions such as AF and PAD. In contrast, the tailored group had a significantly higher proportion of STEMI patients, whereas stable angina cases were more frequently managed with a no tailored strategy. Almost all patients (486/490, 99.2%) received aspirin as part of dual antiplatelet therapy (DAPT) at baseline and continued it throughout the follow-up unless contraindicated. Data related to aspirin response were reported in Table 2, but no variation in aspirin dosage was performed on the basis of PFT. A small group of patients (*n* = 75) submitted to tailored therapy underwent ADP10-PA control. This group of patients was represented by 48 patients in clopidogrel, 24 patients in ticagrelor, and 3 patients in prasugrel. After adjustment of therapy in the tailored group we observed a significant variation in ADP10-PA test: in this group, before tailored treatment, we had a median value of ADP of 53.9%, after tailored treatment with a median value of 46.7% (*p* = 0.01). Considering overall population, we had 250 patients in clopidogrel, 69 patients in ticagrelor, and 16 patients in prasugrel; after treatment, we had 215 patients in clopidogrel, 96 patients in ticagrelor, and 24 patients in prasugrel.

### Clinical outcomes

**Fig. 1:**
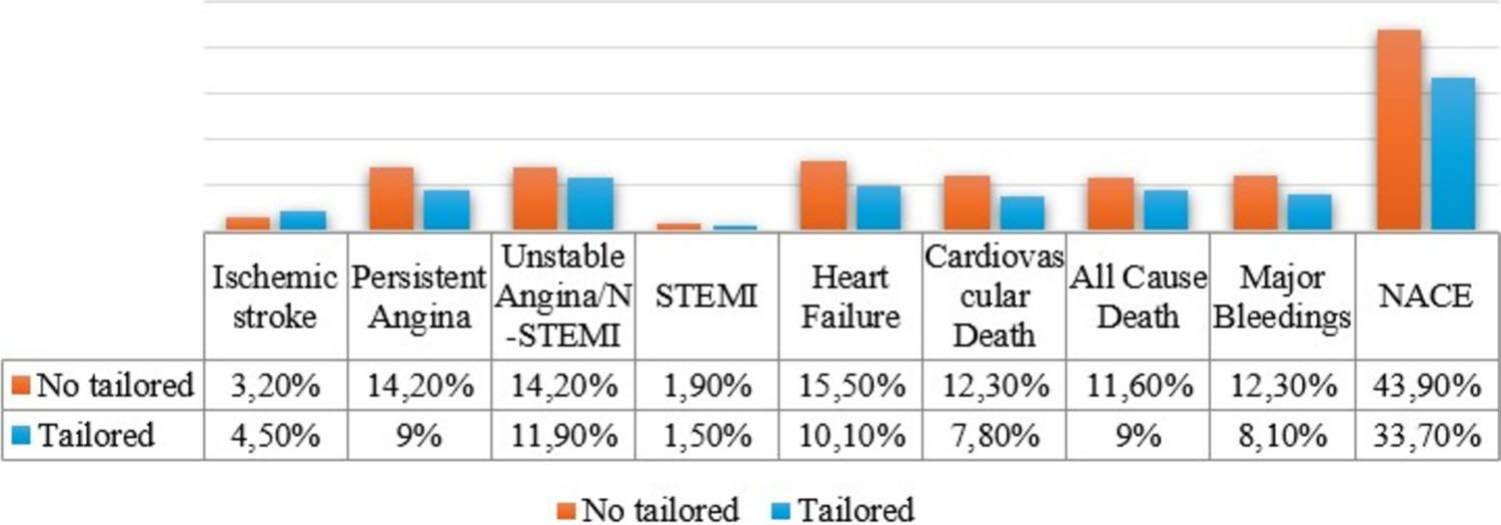
Differences in cardiovascular events between no tailored therapy group and tailored therapy group. *N-STEMI* Non-ST-segment elevation myocardial infarction, *STEMI* ST-segment elevation myocardial infarction, *NACE* Net adverse cardiovascular events

Patients in the tailored therapy group had a significantly lower incidence of NACE at five years (33.7% vs. 43.9%, *p* = 0.02) (Fig. 1). The reduction in ischemic events was accompanied by a lower rate of major bleeding events (8.1% vs. 12.3%, *p* = 0.03). Importantly, patients with high platelet reactivity who were switched to clopidogrel experienced fewer bleeding complications without an increase in ischemic outcomes (Table 3).

**Table 3:** Clinical, procedural, biochemical, and follow-up data comparison between no NACE and the NACE group

Variable	No NACE (*n* = 309)	NACE (*n* = 181)	*P* value
Age, mean (SD), y	67.6 (± 11)	73.5 (± 11.3)	0.7
Sex, No. (%)			
Male, No. (%)	251 (81.2%)	129 (71.3%)	0.01
Female, No. (%)	58 (18.8%)	52 (28.7%)	0.01
BMI, mean (SD), Kg/m2	26.2 (± 4.1)	26 (± 4.5)	0.5
Cardiac risk factors			
Family history of CAD, No. (%)	94 (30.6%)	45 (24.9%)	0.1
Former smoker, No. (%)	229 (74.1%)	117 (64.6%)	0.1
Diabetes Mellitus, No. (%)	69 (22.3%)	54 (29.8%)	0.04
Arterial Hypertension, No. (%)	217 (70.2%)	139 (76.8%)	0.07
Hypercholesterolemia, No. (%)	197 (63.8%)	123 (68%)	0.2
Obesity, No. (%)	45 (14.6%)	27 (14.9%)	0.5
AF, No. (%)	19 (6.1%)	27 (14.9%)	0.001
PAD, No. (%)	35 (11.3%)	36 (19.9%)	0.007
CKD, No. (%)	17 (56%)	29 (16.3%)	< 0.001
Previous AMI, No. (%)	57 (18.5%)	43 (239%)	0.09
Previous PCI, No. (%)	68 (22%)	50 (27.2%)	0.09
Previous CABG, No. (%)	13(4.2%)	13 (7.2%)	0.1
LVEF, mean (SD), %	51.4% (± 9.6)	50,9 (± 9.9)	0.1
Patient’s presentation			
Unstable angina, No. (%)	76 (24.7%)	53 (29.3%)	0.01
N-STEMI, No. (%)	64 (20.8%)	55 (30.4%)	0.01
STEMI, No. (%)	105 (34.1%)	40 (22.1%)	0.01
Stable Angina, No. (%)	63 (20.5%)	33 (18.2%)	0.01
Arterial access			
Radial, No. (%)	244 (79%)	142 (785%)	0.5
Femoral, No. (%)	65 (21%)	39 (21.5%)	0.5
Antiplatelet therapy before/in Cath Lab, No. (%)	21 (6.8%)	30 (16.6%)	< 0.001
Left Main disease, No. (%)			
Treated vessels	198 (64.1%)	1146 (64.1%)	0.9
1, No. (%)	84 (27.2%)	48 (26.5%)	0.9
2, No. (%)	27 (8.7%)	17 (9.4%)	0.9
≥ 3, No. (%)			
Implanted stents	8 (2.6%)	8 (4.5%)	
0, No. (%)	160 (51.8%)	70 (38.6%)	0.07
1, No. (%)	77 (25.2%)	57 (31.8%)	0.07
2, No. (%)	64 (20.7%)	46 (25.1%)	0.07
≥ 3, No. (%)	31 (± 19)	37,1 (± 22.6)	0.01
Total length of implanted stents, mean (SD), mm	165 (54.1%)	91 (51.1%)	0.2
Complete revascularization, No. (%)	156 (63.9%)	71 (59.2%)	0.3
Blood tests			
Hb at admission, mean (SD), g/dl	13.7 (± 1.8)	12.9 (± 1.8)	0.2
Post-operative Hb (lowest), mean (SD), g/dl	12.2 (± 1.8)	11.2 (± 2.1)	0.001
Platelets ad admission, mean (SD), n/μl	212,500 (± 58,189)	218,460 (± 84,999)	0.02
Post-operative platelets (lowest), mean (SD), n/μl	178,660 (± 49,852)	175,720 (± 63,414)	0.3
eGFR, mean (SD), dl/min	76.5 (± 39.7)	71.7 (± 46)	0.2
INR, mean (SD)	1.2 (± 0.3)	1.4 (± 0.7)	< 0.001
Upgrade to ticagrelor, No. (%)	23 (7.4%)	21 (11.6%)	0.08
Upgrade to prasugrel, No. (%)	15 (4.9%)	3 (1.7%)	0.06
Downgrade to clopidogrel, No. (%)	3 (1%)	2 (1.1%)	0.9
DAT at discharge, No. (%)	24 (7.8%)	19 (10.5%)	0.1
5-year events			
Persistent Angina, No. (%)	40 (12.9%)	12 (6.6%)	0.02
Heart Failure, No. (%)	22 (7.1%)	36 (19.9%)	< 0.001
All-Cause Death, No. (%)	7 (2.3%)	41 (22.7%)	< 0.001
Tailored Therapy, No. (%)	222 (71.8%)	113 (62.4%)	0.02

*BMI* Body mass index, *CAD* Coronary artery disease, *AF* Atrial fibrillation, *PAD* Peripheral artery disease, *CKD* Chronic kidney disease, *AMI* Acute myocardial infarction, *PCI* Percutaneous coronary intervention, *CABG* Coronary artery bypass grafting, *LVEF* Left ventricular ejection fraction, *N-STEMI* Non-ST-segment elevation myocardial infarction, *STEMI* ST-segment elevation myocardial infarction, *eGFR* estimated glomerular filtration rate, *INR* International normalized ratio, *HPA* High platelet reactivity, *DAT* Dual antithrombotic therapy (oral anticoagulation and antiplatelet therapy), *DAPT* Dual antiplatelet therapy, *NACE* Net adverse cardiovascular events

Furthermore, our analysis revealed statistically significant differences in the incidence of NACE among various subgroups. Specifically, female patients (28.7% vs. 18.8%, *p* = 0.01), those with AF (14.9% vs. 6.1%, *p* = 0.001), PAD (19.9% vs. 11.3%, *p* = 0.007), and chronic kidney disease (16.3% vs. 5.6%, *p* < 0.001) experienced a higher rate of adverse events. Patients with N-STEMI and UA showed a higher incidence of NACE compared to those with STEMI. In addition, the presence of left main disease (16.6% vs. 6.8%, *p* < 0.001), a greater total stent length, and lower hemoglobin (Hb) levels were also significantly associated with an increased risk of NACE. In the first 12 months after PCI about 17% of patients treated with ticagrelor or prasugrel underwent a downgrade to clopidogrel in both groups, in a variable time frame for side effects or intolerance. Also after the removal of these patients from the analysis no significant difference between antiplatelet regimen and NACE at follow-up was observed (40.6% vs. 31.2%, *p* = 0.02).

### Multivariate analysis

**Fig. 2:**
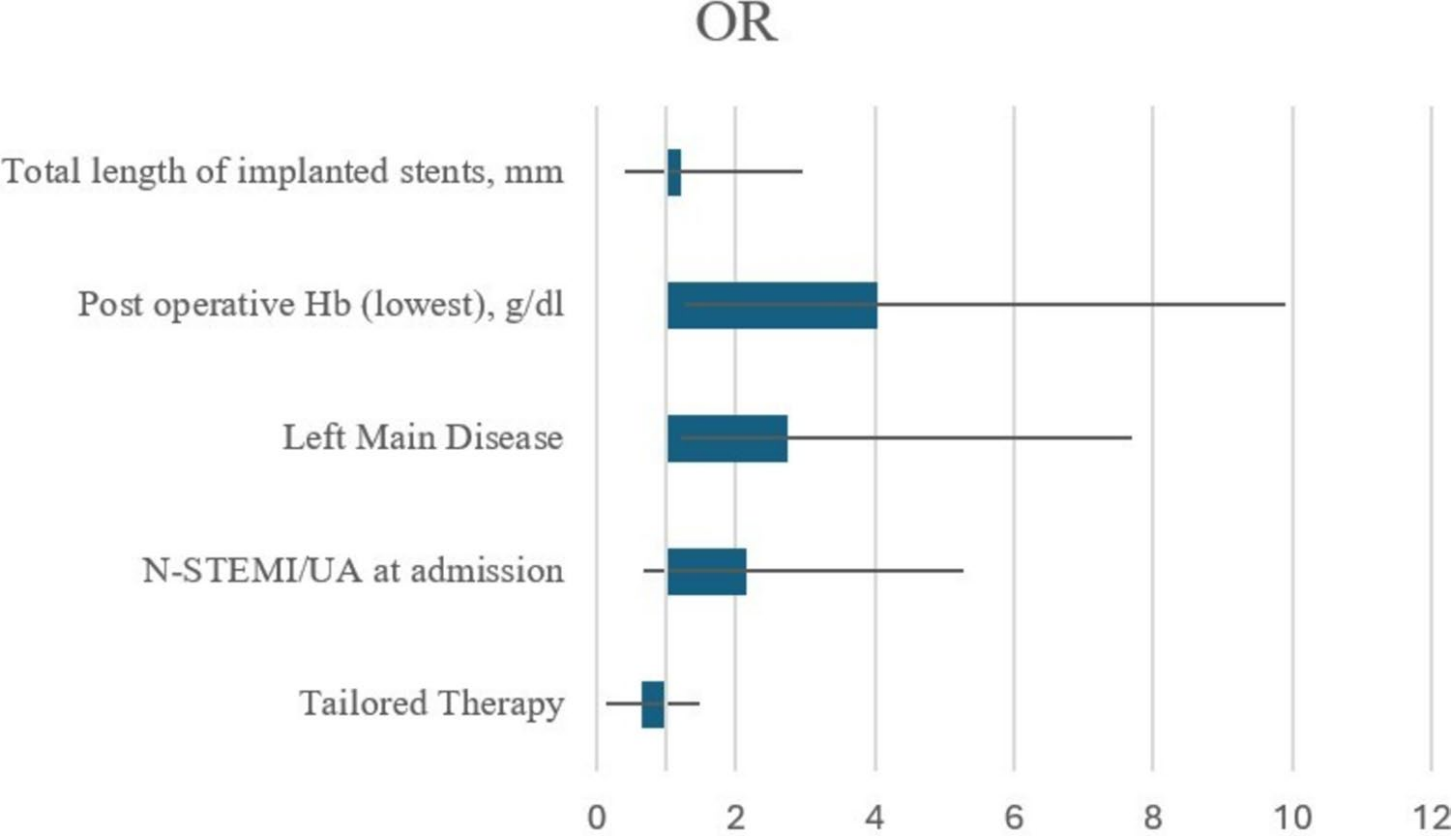
Forest Plot of Multivariate analysis exploring the main predictors of NACE. *OR* Odds ratio, *Hb* Hemoglobin, *N-STEMI* Non-ST-segment elevation myocardial infarction, *UA* Unstable angina

Multivariate regression analysis identified several independent predictors of adverse outcomes (Fig. 2). Total stent length was significantly associated with higher adverse event rates (OR 1.2, CI 95% 0.8–1.8, *p* = 0.01), as well as Hb nadir during hospitalization (OR 4, CI 95% 2.8–5.9, *p* < 0.001). Patients with left main disease had a higher risk of events (OR 2.7, CI 95% 1.5–5, *p* = 0.01), and those presenting with N-STEMI and UA were also at greater risk (OR 2.1, CI 95% 1.5–3.1, *p* = 0.03). Tailored therapy was protective against NACE (OR 0.7, CI 95% 0.5–0.8, *p* = 0.03) and remained independently associated with NACE even after adjustment for AF, PAD, and CKD (*p* = 0.04).

## Discussion

This study underscores the importance of personalized antithrombotic therapy in patients undergoing PCI, particularly in balancing the dual risks of ischemia and bleeding. The five-year follow-up demonstrates that tailored therapy based on platelet function testing significantly reduces adverse outcomes when compared to standard therapy. These findings align with recent studies that emphasize the benefits of individualized treatment strategies in reducing both ischemic and bleeding events in high-risk populations [15, 16].

### Impact of tailored therapy

Our results showed a 10% absolute reduction in NACE at five years, with a favorable safety profile, particularly regarding bleeding complications. This is consistent with findings from the TROPICAL-ACS [17] and POPular Genetics [10] trials, both of which highlighted the value of tailoring antiplatelet therapy based on platelet reactivity in reducing adverse cardiovascular events. Moreover, these trials have highlighted that incorporating genetic testing, particularly for CYP2C19 polymorphisms, can improve personalized antiplatelet therapy. Loss-of-function polymorphisms in CYP2C19 significantly impact clopidogrel’s antiplatelet efficacy, increasing the risk of thrombotic events in patients with this genetic profile. Therefore, either platelet reactivity or genetic testing has proven beneficial in guiding the choice between clopidogrel and more potent inhibitors such as ticagrelor and prasugrel, leading to better clinical outcomes and lower bleeding risks in tailored therapies [10, 17]. The ability to adjust therapy based on platelet function testing, especially in patients with HPR, may explain the reduced bleeding risk without a significant increase in ischemic events. Current guidelines from major cardiology societies, including the ACC and ESC, emphasize the importance of personalized strategies in managing antiplatelet therapy in patients undergoing PCI, particularly those at high bleeding risk [18]. Platelet function and genetic testing are recommended tools to guide such approaches. Our findings align with these recommendations, demonstrating that tailored antiplatelet therapy based on platelet reactivity and risk profiles significantly improves clinical outcomes in patients submitted to PCI [18].

Nevertheless, while our study employed a PFT-guided approach, other strategies have also demonstrated clinical benefits.

The HOST-REDUCE POLYTECH-ACS trial investigated a prasugrel-based dose de-escalation strategy in patients with ACS undergoing PCI [19]. After one month of standard-dose prasugrel (10 mg daily), patients were randomized to continue the same dose or reduce to 5 mg daily without guidance from PFT or genetic testing. The de-escalation group experienced a significant reduction in NACE at one year, primarily driven by a decrease in bleeding events, without an increase in ischemic events.

Similarly, the TALOS-AMI trial evaluated an unguided de-escalation strategy by switching from ticagrelor to clopidogrel one month after PCI in stabilized patients with acute myocardial infarction [20]. The de-escalation group showed a lower incidence of the composite endpoint of cardiovascular death, myocardial infarction, stroke, and bleeding events compared to the group that continued ticagrelor, with the benefit mainly attributed to reduced bleeding.

In our analysis, there was no significant difference in the percentage of changes in P2Y12 inhibition therapy between tailored and no tailored group; one factor possibly explaining this phenomenon is that in the no tailored group upgrade of therapy was performed on the basis of patients complexity/high thrombotic risk, while in the tailored group this upgrade was not performed after clopidogrel responsiveness demonstration; this consideration could also help explain the reduction of hemorrhagic events in this latter cohort.

Unlike unguided de-escalation strategies tested in trials, our study supports a clinically driven, individualized adjustment of antithrombotic therapy based on bleeding risk, ischemic burden, and procedural complexity.

### Predictors of adverse outcomes

The identification of key predictors of adverse events, such as total stent length and left main disease, highlights the complexity of managing PCI patients. A greater stent length is often considered a surrogate marker for more extensive and complex coronary artery disease [21]. It reflects a diffuse atherosclerotic burden and directly predisposes patients to higher risks of stent thrombosis and restenosis, likely due to increased endothelial damage and subsequent neointimal proliferation [22, 23]. Moreover, longer treated lesions may increase the likelihood of incomplete revascularization, which can result in residual ischemia and poorer long-term clinical outcomes [24]. Left main disease is often associated with more advanced and diffuse coronary pathology, further compounding the risk; additionally, left main disease is well known for its higher risk profile, with incomplete revascularization or suboptimal stent placement contributing to worse outcomes, as pointed by the SYNTAX trial and subsequent analyses [25, 26].

Low hemoglobin levels were another critical predictor of adverse outcomes. Anemia is a well-recognized risk factor for both ischemic and bleeding complications, especially in the setting of ACS or PCI [27–30]. Several studies have demonstrated that anemia increases mortality, likely due to reduced oxygen delivery to ischemic myocardium, as well as an increased likelihood of bleeding events [27, 28]. The management of anemia in PCI patients, including the potential role of blood transfusions and iron supplementation, remains an area of active research [29, 30].

In our multivariate analysis, the combined category of N-STEMI and UA emerged as an independent predictor of adverse outcomes. Interestingly, while STEMI patients are often perceived as the highest-risk group in the acute phase, our data suggest that patients with N-STEMI/UA may actually experience a worse long-term prognosis. This observation could be explained by the fact that patients in this category are generally older and tend to have a higher burden of comorbidities, such as AF, PAD, and chronic kidney disease, which collectively contribute to poorer outcomes [31–33]. Moreover, the pathophysiology of N-STEMI and UA, often characterized by diffuse coronary artery disease and more subtle clinical presentations, might result in less aggressive revascularization strategies or residual ischemia over time [32, 33]. These findings underscore the importance of comprehensive risk stratification beyond the initial clinical presentation, as tailoring antithrombotic therapy based on individual risk profiles remains crucial for optimizing long-term outcomes.

### Clinical implications and future directions

The findings of this study could have important clinical implications. They suggest that the routine use of platelet function testing could guide more personalized antithrombotic therapy, leading to a reduction in both ischemic and bleeding events. However, larger, multi-center, and randomized studies are needed to confirm these results and to assess the long-term safety of such strategies. Furthermore, the integration of genetic testing, such as CYP2C19 polymorphisms, with platelet function testing may offer additional benefits in optimizing therapy [33–35].

In addition, the role of newer P2Y12 inhibitors and strategies for de-escalation of antiplatelet therapy, particularly in patients at high bleeding risk, should be further explored. The findings of published trials like TWILIGHT and SMART-DATE have already demonstrated the safety and efficacy of switching to ticagrelor monotherapy after a brief period of dual antiplatelet therapy, significantly reducing bleeding risks without increasing ischemic events [36, 37]. The more recent guidelines recommend for STEMI and N-STEMI patients the use of Prasugrel and Ticagrelor [18]; future research will assess the role of a tailored therapy in patients with chronic coronary syndromes, especially in the presence of multivessel coronary disease and left main involvement, which is a field not completely explored yet.

Another potential field of application for tailored antithrombotic therapy is in patients with AF requiring long-term anticoagulation: these patients typically receive a combination of oral anticoagulants and a single antiplatelet agent, most commonly clopidogrel, to balance the competing risks of ischemic and bleeding events. However, platelet reactivity varies significantly among individuals, and a tailored approach based on PFT could help refine the selection of antiplatelet therapy, optimizing safety and efficacy. Future studies should explore whether adjusting antiplatelet therapy in this high-risk population can further reduce thrombotic complications while minimizing hemorrhagic risks.

## Conclusion

Tailored antithrombotic therapy based on PFT can reduce long-term adverse outcomes in patients undergoing PCI. Personalized therapy could allow for a more balanced approach to managing the dual risks of ischemia and bleeding, especially in high-risk populations such as those with ACS or left main disease. Future studies should focus on integrating genetic testing and expanding personalized treatment strategies to optimize long-term outcomes.

## Limitations

The study’s main limitations are its single-center and retrospective design, which does not allow to draw definitive conclusions for the presence of potential bias. Moreover, it lacks genetic testing data, an information that could have helped the personalization of therapy. Additionally, reliance on PFT alone may overlook other influential factors, and the retrospective nature of the study and the absence of standardized criteria for patient referral to PFT may introduce selection bias.

## Data Availability

The datasets generated and analyzed during the current study are available from the corresponding author upon reasonable request. Due to privacy and ethical restrictions, the data are not publicly accessible.
